# Uncoupling Protein 2 and Nitric Oxide Deficiency Favor Glycolytic Pathways and Organ Growth in the Rat Spleen

**DOI:** 10.3390/cimb48080775

**Published:** 2026-07-30

**Authors:** Lea Wagner, Rolf Schreckenberg, Nadja Itani, Tsuneshiro Sato, Julia Sperhake, Yva Cesar, Klaus-Dieter Schlüter

**Affiliations:** 1 Physiologisches Institut, Justus-Liebig-Universität, 35392 Gießen, Germany; lea.wagner@med.uni-giessen.de (L.W.); rolf.schreckenberg@physiologie.med.uni-giessen.de (R.S.); nadja.itani@physiologie.med.uni-giessen.de (N.I.); sato_tsune@outlook.jp (T.S.); julia.m.sperhake@med.uni-giessen.de (J.S.); yvi.cesar@googlemail.com (Y.C.); 2 Department of Anesthesiology and Intensive Care, Hamamatsu University of Medicine, Hamamatsu 431-3192, Japan

**Keywords:** PDHA1, superoxide radicals, splenomegaly, IL6

## Abstract

Uncoupling protein 2 (UCP2) is expressed in various tissues throughout the body, but its expression in the spleen exceeds that of other organs. However, the precise function of UCP2 for spleen physiology is unclear. The spleen acts as a hub connecting the nervous system and immune system to cardiovascular and metabolic diseases. Here, we analyzed the impact of hypertension on the spleen and the role of UCP2 in this process. Experiments were performed with UCP2-knockout rats and their wild-type littermates. Hypertension was induced by administering the nitric oxide inhibitor L-NAME via tap water. Genetic depletion of UCP2 increased spleen size (splenomegaly) and strongly impaired the expression of genes coding for mitochondrial proteins. Among them, genes coding for proteins involved in oxidative metabolism, such as pyruvate dehydrogenase alpha 1, and the detoxification of reactive oxygen species were down-regulated. Collectively, these alterations in metabolism favor glycolysis and proliferation. Moreover, NOS3 was among the strongest down-regulated genes in UCP2^−/−^ rats, and the inhibition of nitric oxide synthase by L-NAME mimicked large parts of the expression profile. Neither the depletion of UCP2 nor L-NAME-induced hypertension or combinations thereof affected chronic inflammation. In summary, UCP2 controls fuel consumption in splenic cells in a nitric-oxide-dependent way.

## 1. Introduction

The spleen has multiple functions in the body. It recognizes and removes old and malformed erythrocytes and plays a role in B-cell maturation. Furthermore, it acts like a hub connecting the nervous and immune systems to cardiovascular and metabolic diseases [1]. Mitochondrial uncoupling protein 2 (UCP2) is differentially expressed in various tissues throughout the body. However, its expression in the spleen exceeds that of all other tissues [2,3,4,5,6]. This uncoupling protein has been identified as a molecule affecting metabolic flux in cells [7]. Former studies suggest that UCP2 is important for the detoxification of oxygen radicals (reviewed in ref. [8]). These assumptions suggest that UCP2 plays an important role in immune and metabolic diseases by modifying the micro-milieu in the spleen. However, UCP2-depleted rats do not have an obvious immune defect or metabolic disorder [9]. In UCP2-knockout mice, an increased insulin sensitivity and reduced inflammation under high-fat diet was described [10,11]. Sterile inflammation is a hallmark of cardiovascular disease [12]. In an angiotensin II-dependent hypertension model, it was shown that immune cells migrate from the spleen to the heart [13]. Therefore, any changes in spleen physiology may affect cardiovascular physiology under hypertensive conditions as well. Here we administered L-NAME (L-N^G^-Nitro arginine methyl ester), an inhibitor of nitric oxide synthases (NOS), to induce hypertension in rats. L-NAME induced a phenotype of right-heart hypertrophy, a condition often associated with splenomegaly [14,15,16]. To identify the role of UCP2 in this context, we compared the response of wild-type rats and UCP2-deficient rats to L-NAME and analyzed the amount of oxidative stress, expression of genes coding for proteins of mitochondria, and inflammation. In summary, the current study aimed to investigate the role of UCP2 in hypertension-induced physiological adaptations of the spleen.

## 2. Materials and Methods

### 2.1. Animal Experiments

The investigations are in agreement with the “Guide for the Care and Use of Laboratory Animals” purchased by the US National Institute of Health (NIH Publication No. 85-23, revised 1996). The studies were approved by the local authorities (RP Gießen; V54–19 c 20 15 h 01 GI 20/1 Nr. G 70/2021).

This study was performed on adult female wild-type (UCP^+/+^) and UCP2-knockout (UCP2^−/−^) rats (4 months old). Housing was performed in standard animal cages (three rats per cage), with 12 h day/night cycle, 21 °C and 40–60% humidity. Rats were randomly put into one of four groups (n = 10 rats per group): UCP2^+/+^ (mean weights at start 235 g, final weight 261 g, Δ26 g), UCP^+/+^ + L-NAME (mean weights at start 248 g, final weight 269 g, Δ21 g), UCP2^−/−^ (mean weights at start 244 g, final weight 271 g, Δ27 g), and UCP2^−/−^ + L-NAME (mean weights at start 252 g, final weight 277 g, Δ25 g). Group sizes were calculated on the basis of blood pressure increases > 20 mmHg. Exclusion criteria were defined as loss of body weight during the four-week experimental period. There were no exclusions in this study. L-NAME was administered via tap water at a concentration of 7.5 mg/day. L-NAME solution (30 mg/80 mL) was prepared daily and provided via tap water. The mean uptake of L-NAME was calculated by tap water consumption resulting in 7.5 mg L-NAME per day (average dose: 30 mg/kg body weight). L-NAME increased blood pressure in WT rats from 117 ± 2 mmHg to 157 ± 5 mmHg, and in KO rats from 124 ± 5 mmHg to 162 ± 3 mmHg (n = 4 each group). Blood pressure was measured by tail-cuff method as described before (ref. [17]). For initial experiments, another group (n = 10) of UCP2^+/−^ rats (female, 12 weeks old) was used for RNA analysis. In total, 56 rats (either UCP^+/+^, UCP2^+/−^, or UCP2^−/−^) were used in this study.

To finalize all experiments, rats were weighted and subsequently anesthetized by isoflurane inhalation. After cervical dislocation, spleens and tibia were separated. Spleens were weighed, and tibia lengths were measured. Subsequently, tissues were quickly frozen in fluid nitrogen and stored until use at −80 °C.

### 2.2. RT-PCR

Total RNA was isolated from the spleen using peqGold TriFast (peqlab, Biotechnologie GmbH, Erlangen, Germany) according to the manufacturer’s protocol. Genomic DNA contamination was avoided by use of 1 U DNase/µg RNA (Invitrogen, Karlsruhe, Germany). Synthesis of cDNA and subsequent Real-time PCR was performed as described before [17]. A list of primers used in this study can be found in the Supplementary Table S1. For the screening of our samples, we used reference genes for the following pathways: mitochondria (*UCP2*, *UCP3*, *VDAC*, *MPC1*, *MPC2*, *MFN2*, *CPT1B*), oxidative stress (*CAT*, *SOD1*, *SOD2*, *SOD3*, *MAOA*, *MAOB*, *GPX1*), apoptosis (*BNIP3L*, *BCL2*, *PINK1*, *OMA1*, *BAX*, *PARK2*), fatty acid metabolism (*CD36*, *PPARA*, *FABP5*, *HADHA*, *FABP3*), cytoskeletal proteins (*DNM1*, *LAMP2*, *MAP1LC3B*), inflammation (*IL10*, *IL6R*, *IL12A*, *IL6*), glucose metabolism (*PDHA1*, *LDHC*, *SLC2A1*, *SCL2A4*, *PDK1*, *HIF2A*, *HIF1A*), arginine metabolism (*NOS3*, *ARG1*, *ARG2*), and fibrosis (*COL1A1*, *COL3A1*, *FN1*, *LAMA1*).

### 2.3. Western Blot

Protein extracts from tissues were generated as described before [17]. Primary antibodies were directed against AKT (Akt antibody, Cell Signaling, Danvers, MA, USA; #9272), phospho-Akt (Ser473) (Cell Signaling, Danvers, MA, USA; #4060), PDHA1 (Biozol, Eching, Germany; GTX104015), PDK1 (Proteintech, Planegg-Martinsried, Germany; 18262-1-AP); glyceraldehyde 3-phosphate dehydrogenase (GAPDH; Calbiochem, San Diego, CA, USA; CB1001) and anti-UCP2 (gentle gift from Prof. Dr. E. Pohl, Vienna, Austria) as described before [7]. Secondary antibodies (HRP-conjugated) directed against rabbit IgG were purchased from Affinity Biologicals (Ancaster, ON, Canada; P 0448).

### 2.4. Mitochondrial Function

Mitochondria were isolated from spleen tissue. Mitochondrial function was analyzed on the basis of n = 6 additional wild-type and knockout rats. Here, the spleen was immediately minced after preparation with scissors in ice-cold homogenization buffer A (100 mmol/L KCl, 50 mmol/L 3-[N-morpholino]-propane sulfonic acid (MOPS), 5 mmol/L MgSO_4_, 1 mmol/L EGTA, 1 mmol/L ATP, pH 7.4). Then the preparation was homogenized six times with a 15 mL glass potter. The homogenate was centrifuged at 800× *g* for 10 min at 4 °C. The supernatant containing mitochondria was removed and centrifuged again at 8000× *g* for 10 min at 4 °C. The sediment was washed with buffer A (without ATP) and subsequently resuspended in a small volume of the same buffer and stored on ice until use.

The respiration of 0.1 mg/mL mitochondria was measured in a Clarke-type oxygen electrode (Strathklevin, Glasgow, UK) at 25 °C in buffer B (250 mmol/L KCl, 20 mmol/L 3-[N-morpholino]-propane sulfonic acid (MOPS), 2.4 mmol/L KH_2_PO_4_, 2.4 mmol/L MgCl_2_, 40 µmol/L EGTA, pH 7.4). Glutamate (5 mmol/L) and malate (2.5 mmol/L) were used as substrates for complex I and succinate (5 mmol/L) as substrate for complex II, in the presence of rotenone. ADP (40 µmol/L) was added to start respiration.

Mitochondrial ROS production was analyzed as described before [7]. Amplex UltraRed was used as an ROS-sensitive substrate. Glutamate and malate were added to feed the electron transport chain. A Cary Eclipse spectrophotometer (Agilent Technologies, Santa Clara, CA, USA) was used at the excitation wavelength of 565 nm and an emission wavelength of 581 nm.

### 2.5. Histological Staining

Cryosections of the spleen were incubated with dihydroethidium (DHE, D23107, Thermo Fisher Scientific, Dreieich, Germany) dissolved in 1 x phosphate-buffered saline for 10 min at 37 °C in a light-protected humidity chamber, then fixed with Dako Fluorescent Mounting Medium (S3023, Dako, North America Inc., Carpinteria, CA 93013, USA). Slides were imaged by Keyence Microscope (BZ-X800Keyence, Neu-Isenburg, Germany) using 545 nm (excitation wavelength) and 605 nm (emission wavelength). Digital images were analyzed using ImageJ software (Version 1.54 d).

To count the number of cells, nuclei were counted based on classical hematoxylin and eosin staining. Briefly, cryosections were fixed with 4% paraformaldehyde and exposed to Gill’s hematoxylin and subsequent Eosin G-solution.

### 2.6. ELISA

Plasma samples were generated as previously described [12]. In these samples, we analyzed the amount of IL6 and IL10 with a commercial ELISA (Rat IL-6, Biotech, R&D Systems, Besancon, France; R6000B) and IL-10, Merck-Millipore, Darmstadt, Germany; RA00246, respectively). Lactate was quantified in spleen tissues (ELISA: Lactate-Glo^TM^, Promega, Mannheim, Germany).

### 2.7. Statistics

Normal distribution of data was validated by Shapiro–Wilk tests. Normal variance was subsequently analyzed using the Levene test. Subsequently, two group comparisons were made by Student *t*-tests to calculate the *p*-value. Two-sided one-way ANOVA with Student–Newman–Keuls post hoc analysis was used for comparisons of more than two groups. In the figures, *p* < 0.05 is marked. In addition, groups were compared by two-way ANOVA to identify effects of UCP2 deletion that are independent of L-NAME, L-NAME effects that are independent of UCP2 deletion, and interactions between both stressors. These data are given in Supplementary Table S2.

## 3. Results

### 3.1. Expression of UCP2 in the Rat Spleen and Its Genetic Modification

**Figure 1:**
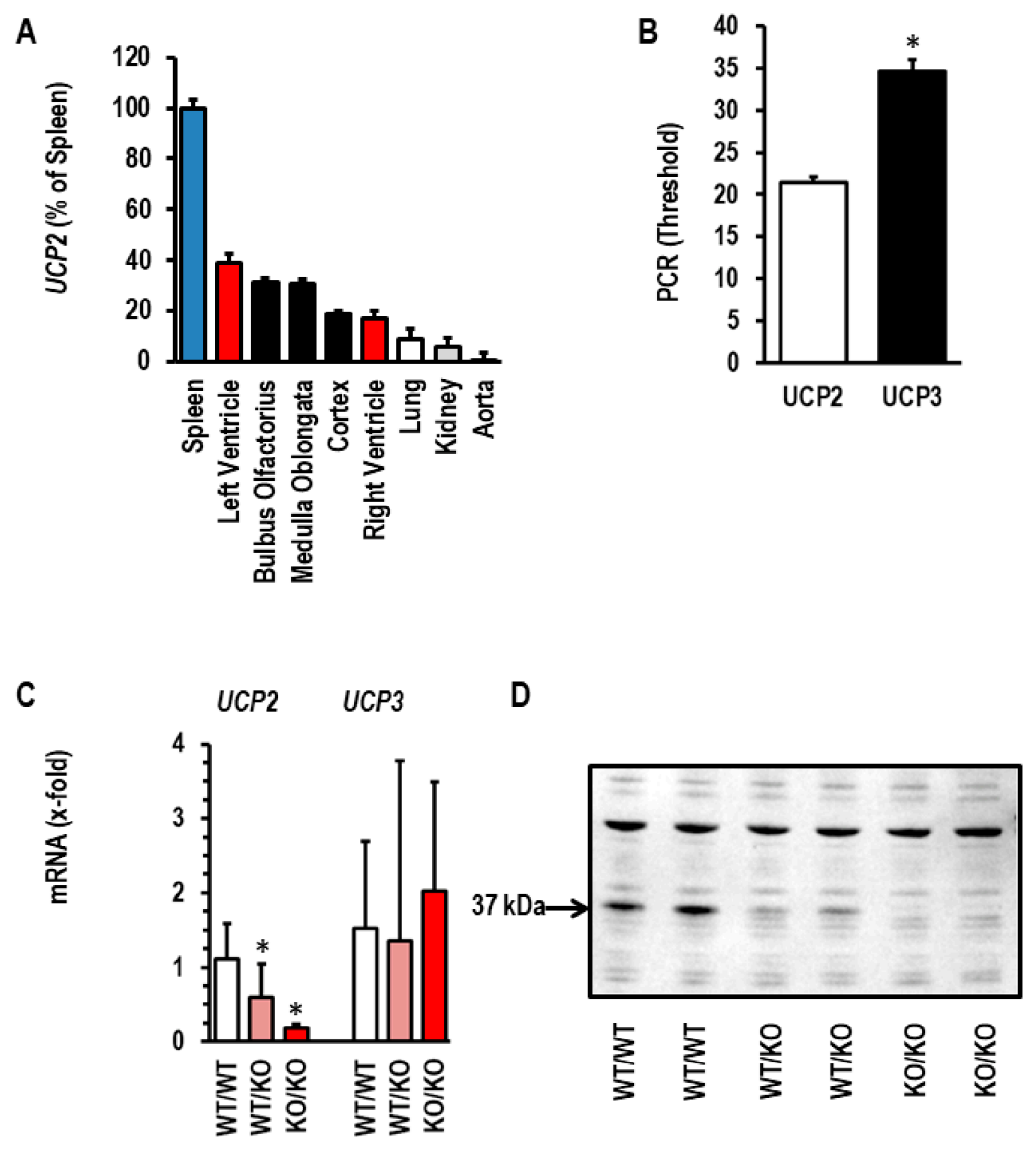
Expression of UCP2 in the rat spleen. (**A**) Gene expression of UCP2 in various tissues of normotensive Wistar rats. The expression (quantified by PCR thresholds) in the spleen was set as 100%. (**B**) Comparison between the expression of UCP2 and UCP3 in spleens from normotensive Wistar rats by PCR thresholds. (**C**) Splenic expression of UCP2 and UCP3 in UCP2-depleted rats (expression in wild-type littermates always set as 1. (**D**) Western Blot indicating the UCP2 protein expression in UCP2-depleted rats. The arrow indicates the UCP2 band at 37 kDa. Data show means ± S.D. from n = 8 rats. *, *p* < 0.05.

The mRNA expression of UCP2 was analyzed in various rat tissues. The expression of UCP2 is strongest in the spleen (Figure 1A). Moreover, gene expression of UCP2 exceeds that of UCP3—another isoform of uncoupling proteins expressed in many rat tissues, including the spleen—because the threshold for UCP2 was much lower than that for UCP3 (Figure 1B). Subsequently, we analyzed whether genetic deletion of UCP2 generates a compensatory up-regulation of UCP3 in the spleen. The data confirmed decreased expression of UCP2 in our knockout rats, but the mRNA expression of UCP3 was not altered (Figure 1C). Therefore, UCP3 does not compensate for UCP2 deficiency in the spleen. Finally, we confirmed that genetic deletion of UCP2 reduces the protein expression of UCP2 (Figure 1D). Collectively, the data show that UCP3 does not compensate for UCP2 depletion and that genetic deletion of UCP2 reduces the protein-level expression of UCP2 in the spleen to levels below the detection level of Western Blot. From these initial experiments, we concluded that the UCP2^−/−^ rat is a suitable model to analyze the role of UCP2 in the adaptation to hypertension.

### 3.2. Effect of Genetic Deletion of UCP2 on the Size of the Spleen

**Figure 2:**
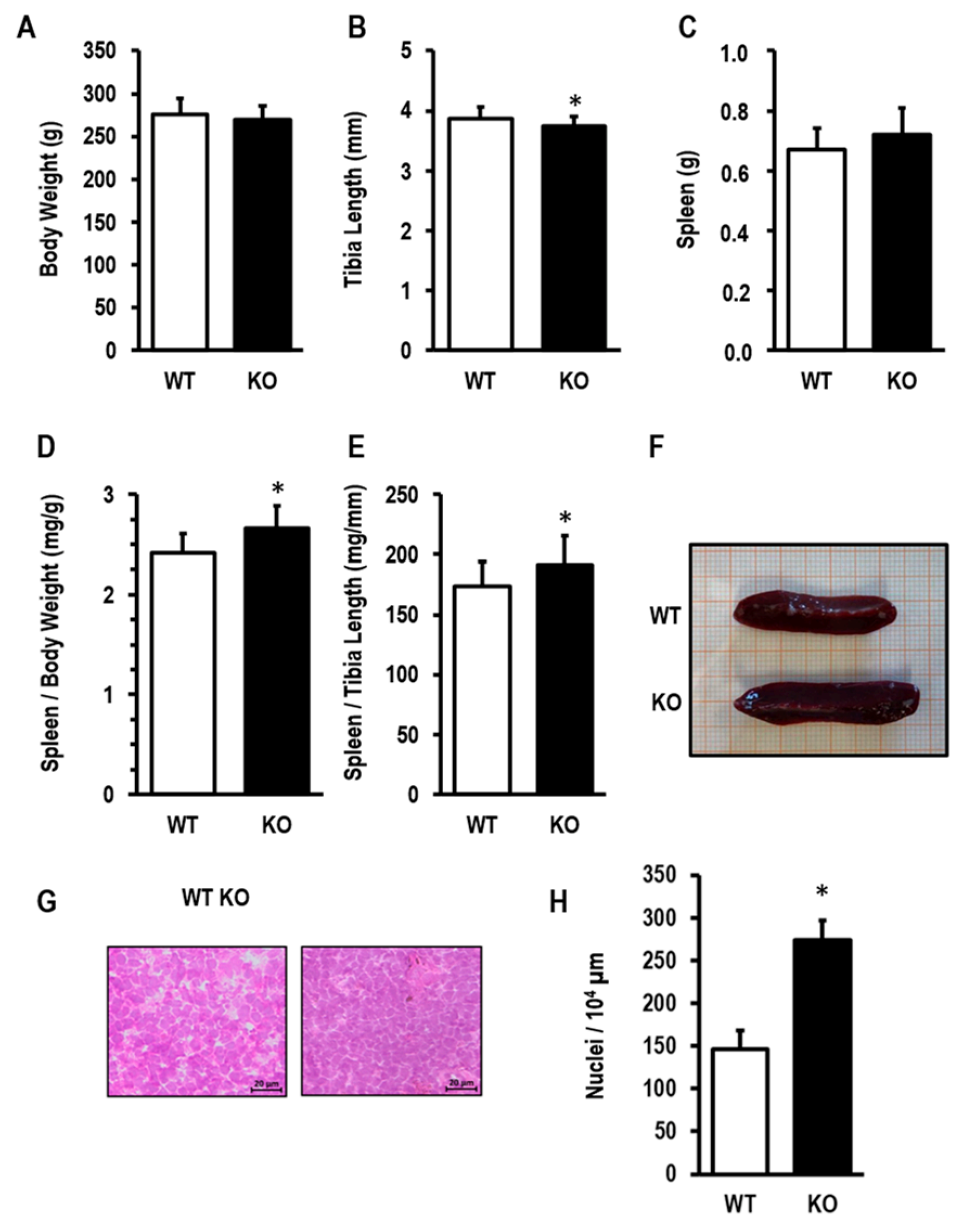
Spleen sizes in UCP2-depleted rats (KO) and wild-type littermates (WT). (**A**) Total body weight; (**B**) tibia length; (**C**) spleen weight; (**D**) spleen weight normalized to body weight; (**E**) spleen size normalized to tibia length; (**F**) original picture of a spleen isolated from WT and KO. (**G**) Histological slices of spleens. (**H**) Quantification of nuclei number. Data are means ± S.D. *, *p* < 0.05 (Student *t*-Test).

Small differences between UCP2^−/−^ rats and UCP2^+/+^ rats were recognized. UCP2^−/−^ had slightly reduced body weight (Figure 2A) and tibia length (Figure 2B). In contrast, the spleen weight was higher (Figure 2C). Upon normalization to body weight (Figure 2D) or tibia length (Figure 2E), UCP2^−/−^ rats had increased spleen sizes. Even macroscopically, the size of the spleen was bigger in KO compared to WT (Figure 2F). A histological analysis of the spleen showed a proliferative phenotype (Figure 2G). A larger magnification of this slight is given in Supplementary Figure S1. Quantitatively, the number of nuclei in UCP2^−/−^ rats exceeded that of WT (Figure 2H).

### 3.3. Mitochondrial Function

**Figure 3:**
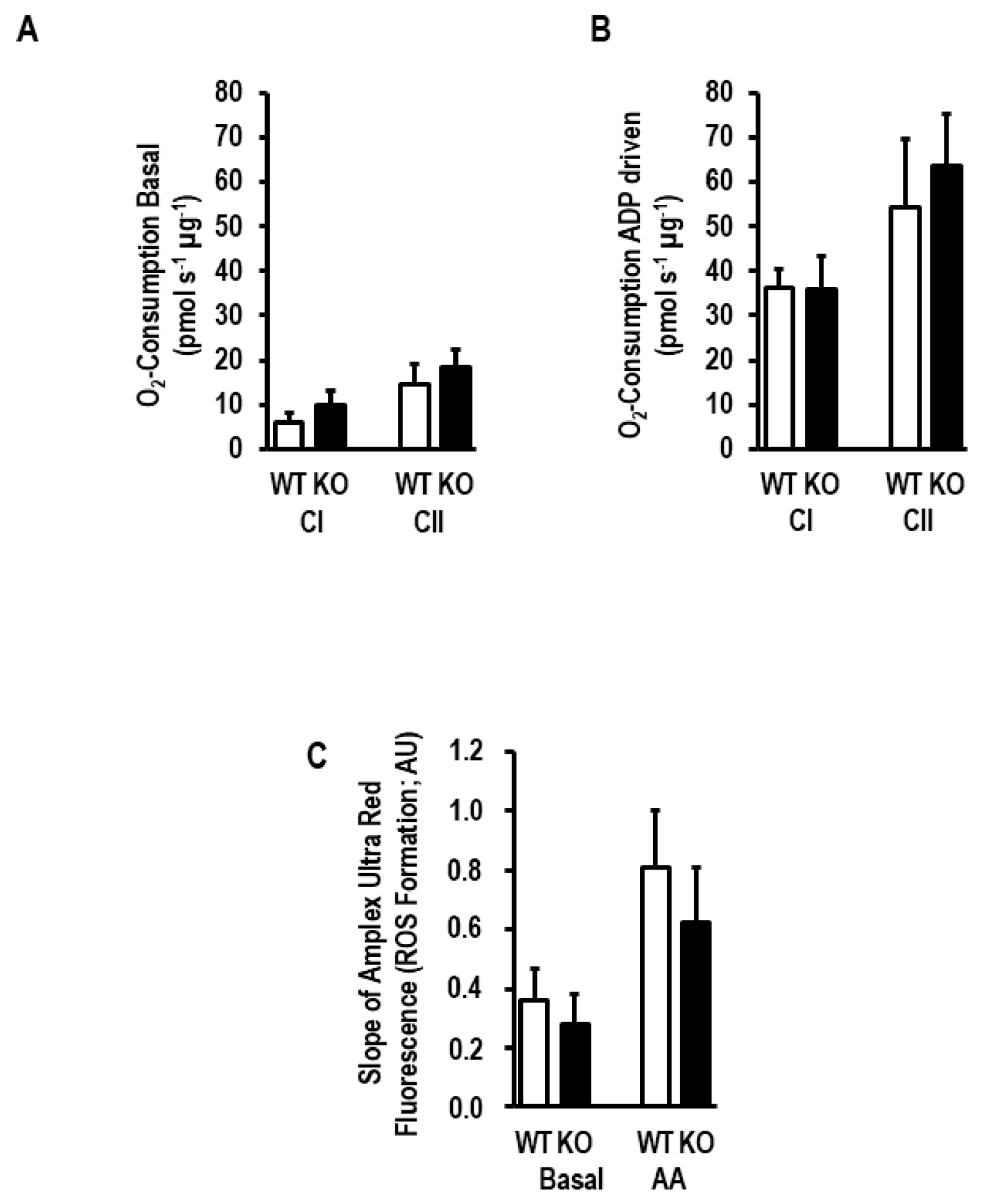
Effect of UCP2 depletion on mitochondrial Function. (**A**) Respirometry from isolated mitochondria from UCP2-depleted rats (KO) and wild-type littermates (WT). (**B**) Respirometry as in A but ADP-driven. (**C**) ROS formation measured in isolated mitochondria from WT and KO rats. Data are means ± S.D. from n = 6 biological replicates. *p* > 0.05 in all cases (Student *t*-Test).

Uncoupling proteins may act as oxygen radical detoxification molecules in mitochondria. As no other UCP isoform is more strongly expressed in the spleen than UCP2, we investigated whether genetic deletion of UCP2 affects oxygen consumption and the mitochondrial production of reactive oxygen species (ROS) in the spleen. However, neither basal oxygen consumption (Figure 3A), ADP-driven oxygen consumption (Figure 3B), nor mitochondrial ROS formation (Figure 3C) showed strong differences between mitochondria function of UCP2^−/−^ and UCP^+/+^. There was at least a trend for increased oxygen consumption, specifically of complex II-dependent oxygen consumption (Figure 3A,B), and a trend for lower mitochondrial ROS formation in UCP2^−/−^ vs. UCP^+/+^ (Figure 3C).

### 3.4. Altered Gene Expression in UCP2-Depleted Rat Spleens

**Figure 4:**
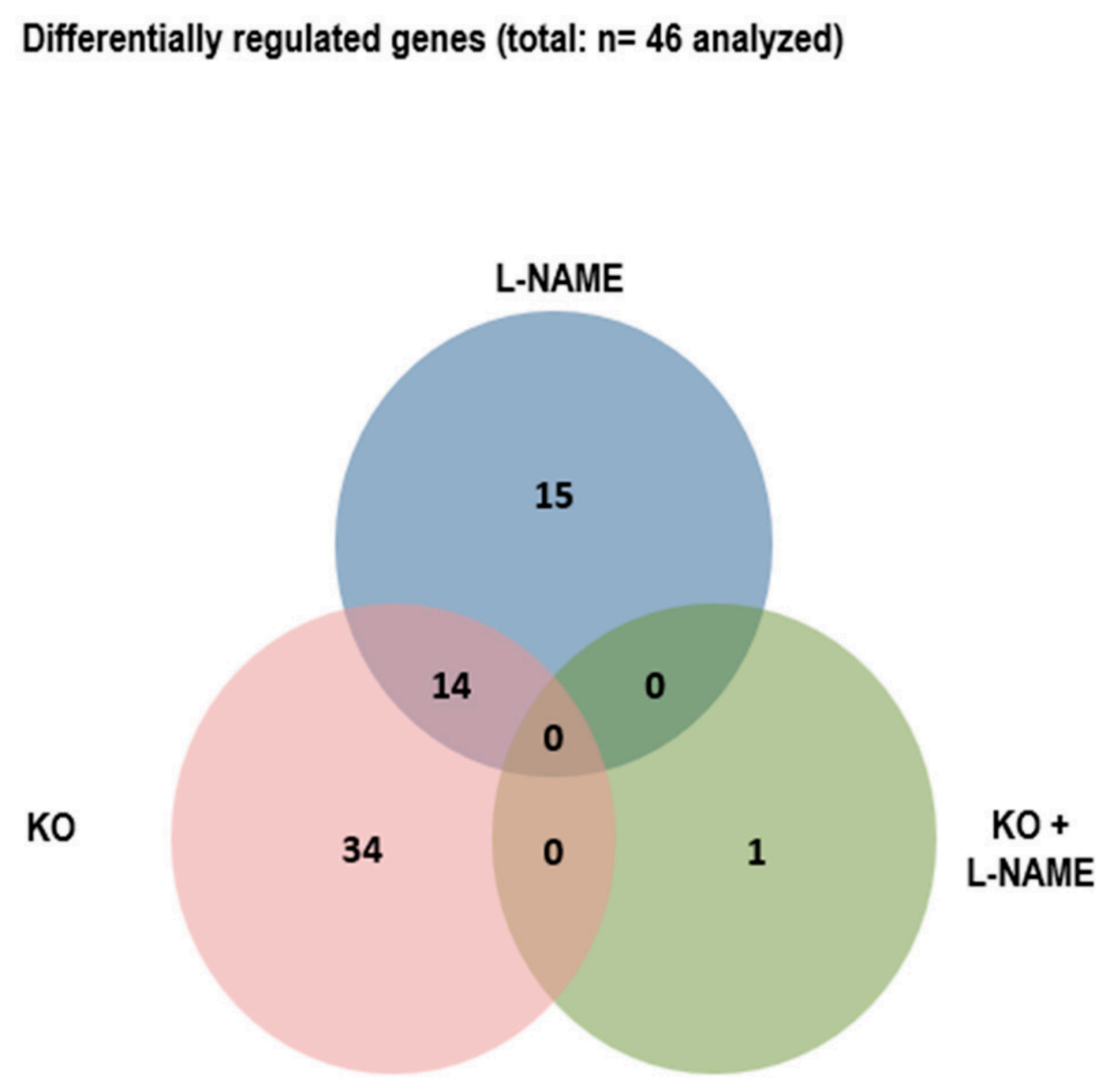
Venn Diagram visualizing the number of differential regulated genes (based on *p*-values below 0.05) between UCP2-depleted rats (KO) and their littermates (UCP2), L-NAME-treated rats and untreated controls (L-NAME), and UCP2-depleted rats treated with L-NAME and untreated UCP2-depleted rats (UCP2/L-NAME).

To identify the role of UCP2 in the spleen, we next analyzed the mRNA expression of forty-six genes that code for proteins linked to mitochondrial function, metabolism, and inflammation. The majority of such genes (thirty-four) was differentially regulated between UCP2^−/−^ and UCP2^+/+^ (Figure 4). Fourteen out of the thirty-four differentially regulated genes were similarly affected when L-NAME was administered to induce hypertension. The combined stress by genetic deletion of UCP2 and hypertension did not further modify gene expression in the spleen (Figure 4). This suggests that genetic deletion of UCP2 mimics a couple of adaptations of the spleen that are also seen under the condition of hypertension.

**Figure 5:**
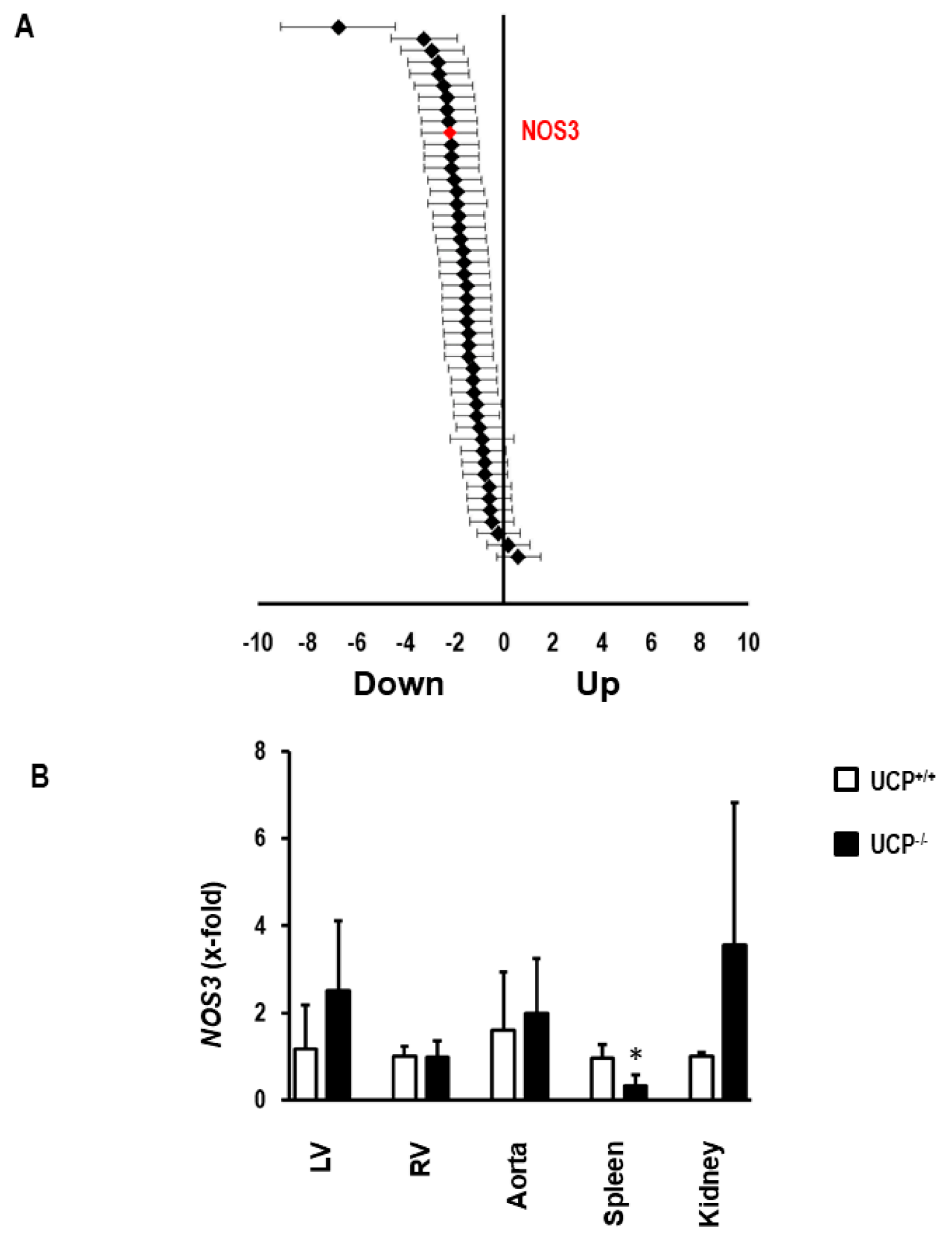
Effect of UCP2 depletion on splenic NOS3 expression. (**A**) Overview of all genes analyzed, indicating NOS3 in red among strong down-regulated genes in UCP2-depleted rats (effect sizes with 25 and 75% confidence intervals). (**B**) Comparison of the effect of UCP2 depletion on NOS3 expression in other tissues (Means ± S.D. from n = 8 biological replicates). *, *p* < 0.05 vs. UCP^+/+^ (Student *t*-Test).

Among the strongest regulated genes by genetic deletion of UCP2 was NOS3 (Figure 5A). The reduced expression of NOS3 in KO rats may give us a hint as to why so many genes are similarly regulated in UCP2-deficient rats and L-NAME-treated wild-type rats. When compared with other tissues, it turns out that the down-regulation of NOS3 in UCP2-depleted rats is rather specific for the spleen (Figure 5B).

### 3.5. UCP2 Depletion and Expression of Mitochondrial Proteins

**Figure 6:**
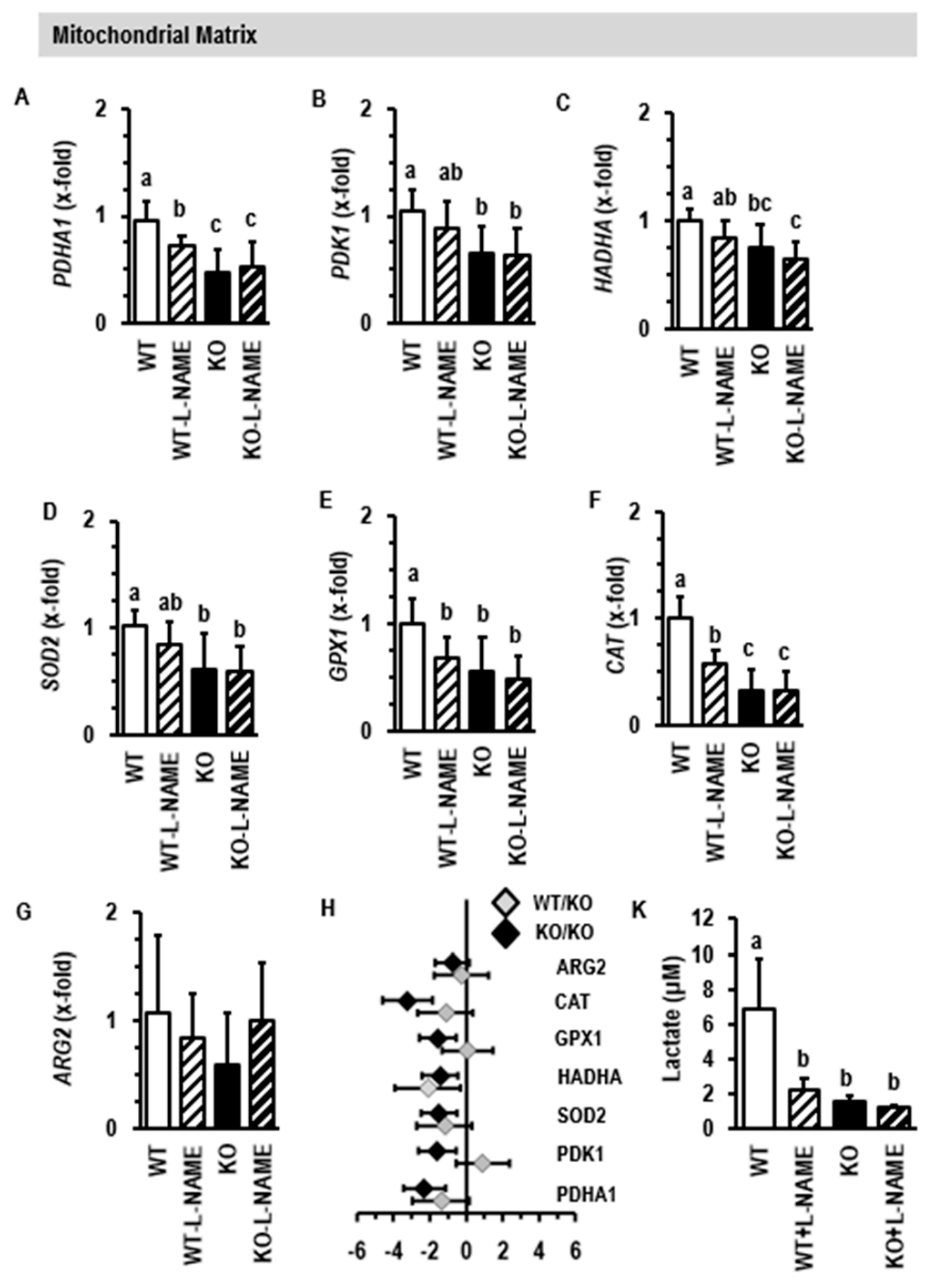
Effect of L-NAME and UCP2 depletion on the expression of genes coding for proteins of the mitochondrial matrix (**A**–**G**). Data are means ± S.D. from n = 10 rats per group. (**H**) Gene dose effect on the regulation of genes by depletion of UCP2. Data are effect sizes with confidence intervals. (**K**) Lactate concentration in spleen tissues (mean ± S.D. from n = 5). Small different letters indicate differences between other groups with *p* < 0.05 (One-way ANOVA with Student–Newman–Keuls post hoc analysis).

The mitochondrial matrix contains several proteins that are important for consumption of either fatty acids, carbohydrates, or both. In addition, proteins are expressed in mitochondria that detoxify ROS. UCP2 is located at the inner mitochondrial membrane of mitochondria. Its depletion in the spleen is associated with reduced expression of *PDHA1*, a gene coding for the enzyme pyruvate dehydrogenase alpha 1 (Figure 6A). This enzyme is part of the pyruvate dehydrogenase complex that links glycolysis with the tricarboxylic acid cycle. Down-regulation of *PDHA1* will therefore reduce oxidative consumption of glucose and improve glycolysis. Similarly, *PDK1*, a gene encoding for the enzyme pyruvate dehydrogenase kinase isoform 1, is also down-regulated in UCP2-depeleted mitochondria (Figure 6B). This enzyme acts as an inhibitor of the pyruvate dehydrogenase complex. Another down-regulated gene in UCP2^−/−^ rats is *HADHA*, coding for the protein trifunctional enzyme subunit alpha (Figure 6C). This protein is part of the mitochondrial trifunctional protein that catalyzes three enzymatic steps in β-fatty acid oxidation. Collectively, these data suggest reduction in oxidative metabolism that favors glycolysis, and vice versa. This phenomenon is known as the “Warburg Effect” or aerobic glycolysis, and it is commonly found in growing tissues like cancers. Furthermore, UCP2 depletion is associated with down-regulation of *SOD2*, *GPX1*, and *CAT* (Figure 6D–F). These genes code for the proteins superoxide dismutase 2, glutathione peroxidase 1, and catalase, which are all required for the detoxification of ROS in mitochondria. In contrast, *ARG2*, a gene coding for the protein arginase 2, was not affected by UCP2 depletion (Figure 6G).

Two-Way ANOVA showed that *PDHA1* and *CAT* were both more strongly affected by the combination of UCP2 deletion and L-NAME (Supplementary Table S2, interaction, *p* < 0.05). Down-regulation of *PDHA1* and *CAT* was more strongly affected in UCP^−/−^ than in UCP2^+/−^ (Figure 6H). Induction of hypertension by administering L-NAME also reduced the expression *PDHA1* and *CAT*, and in both cases the depletion of UCP2 could not further decrease the expression of these genes (Figure 6A–G). Finally, the concentration of lactate was analyzed in spleen tissues (Figure 6K). In all groups with down-regulation of UCP2, we observed less lactate. The data suggest that aerobic glycolysis improves glucose-dependent branch pathways such as the pentose phosphate pathway that generate ribose, which is required for DNA synthesis and serine synthesis and offers NAPDH, a co-factor needed for biosynthesis.

**Figure 7:**
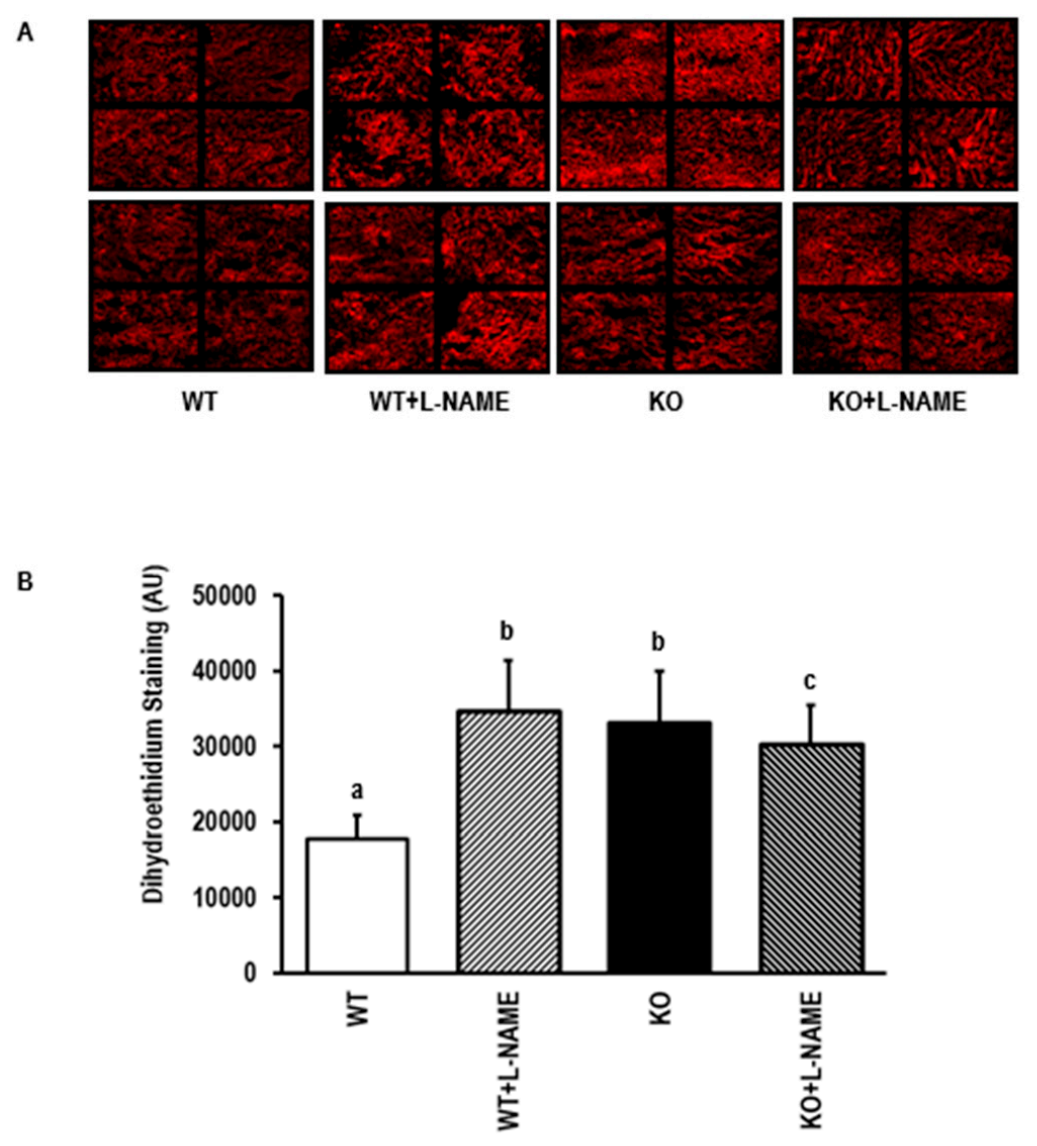
Effect of L-NAME and UCP2 depletion on superoxide formation in the spleen. (**A**) Original pictures of dihydroehtidium staining. (**B**) Quantification of this analysis. Data are means ± S.D. from n = 8 per group. Small different letters indicate differences between other groups with *p* < 0.05 (one-way ANOVA with Student–Newman–Keuls post hoc analysis).

The data further suggest oxidative stress by down-regulation of UCP2 and NO deficiency. This assumption was validated by superoxide quantification (Figure 7A,B). The data indeed showed increased oxidative stress under such conditions, with no additive effect by UCP2 depletion or L-NAME. Two-way ANOVA identified genotype and treatment as independent factors inducing superoxide formation (Supplementary Table S2).

**Figure 8:**
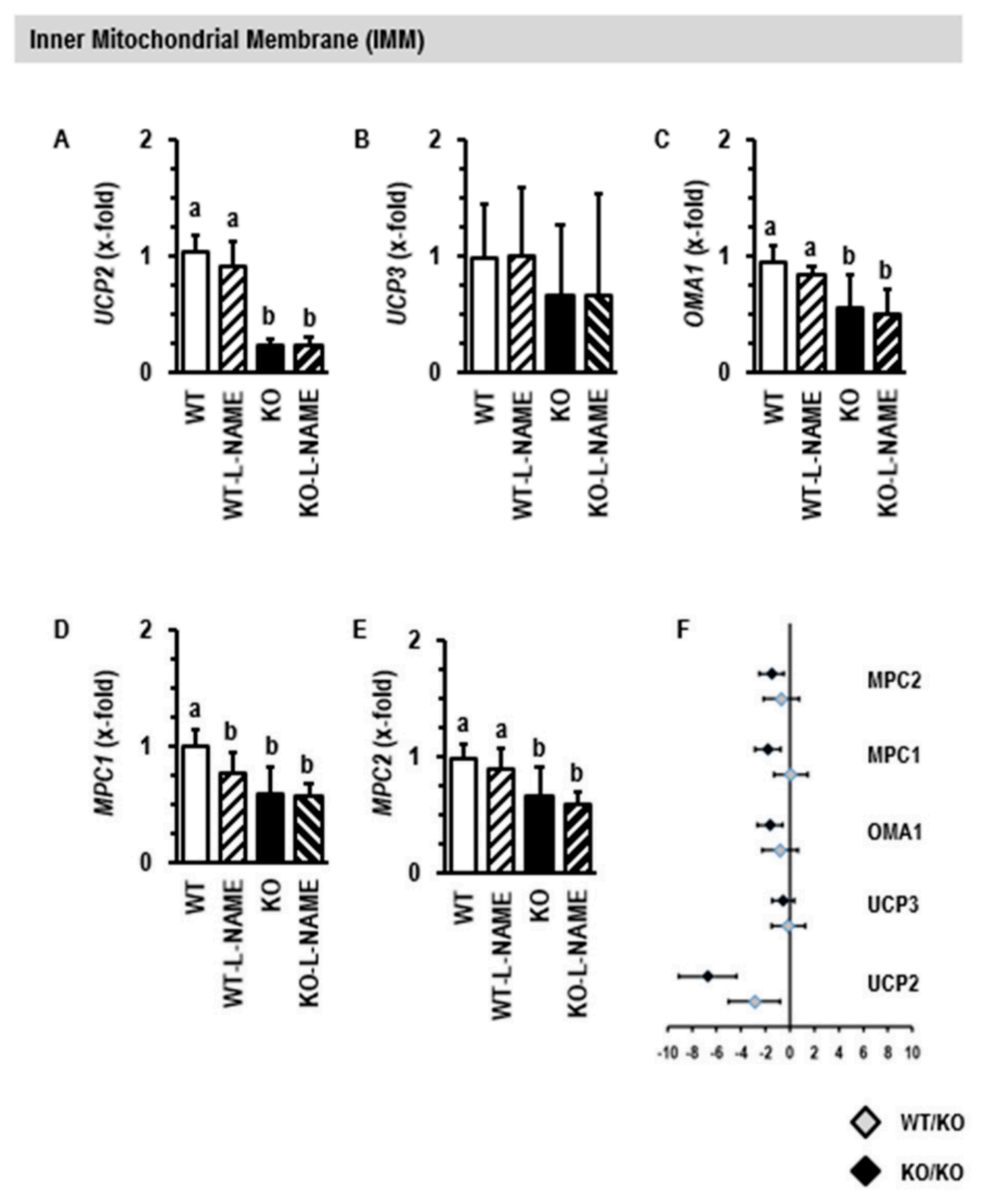
Effect of L-NAME and UCP2 depletion on the expression of genes coding for proteins of the inner mitochondrial membrane (**A**–**E**). Data are means ± S.D. from n = 10 rats per group. (**F**) Gene dose effect on the regulation of genes by depletion of UCP2. Data are effect sizes with confidence intervals. Small different letters indicate differences between other groups with *p* < 0.05 (one-way ANOVA with Student–Newman–Keuls posthoc analysis).

UCP2 is in the inner mitochondrial membrane. L-NAME treatment alone did not affect the expression of UCP2 (Figure 8A). Neither the depletion of UCP2 nor L-NAME affected the expression of the second isoform of UCP expressed in the spleen, namely UCP3 (Figure 8B). *OMA1*, a gene coding for the metalloendopeptidase OMA1, and genes coding for mitochondrial pyruvate carrier 1 and 2 were down-regulated in UCP2-depleted rats (Figure 8C–E). Two-way ANOVA identified *MPC1* as a gene that is also affected by L-NAME alone (Supplementary Table S2). All these changes were stronger in UCP2^−/−^ than in UCP2^+/−^ rats (Figure 8F).

**Figure 9:**
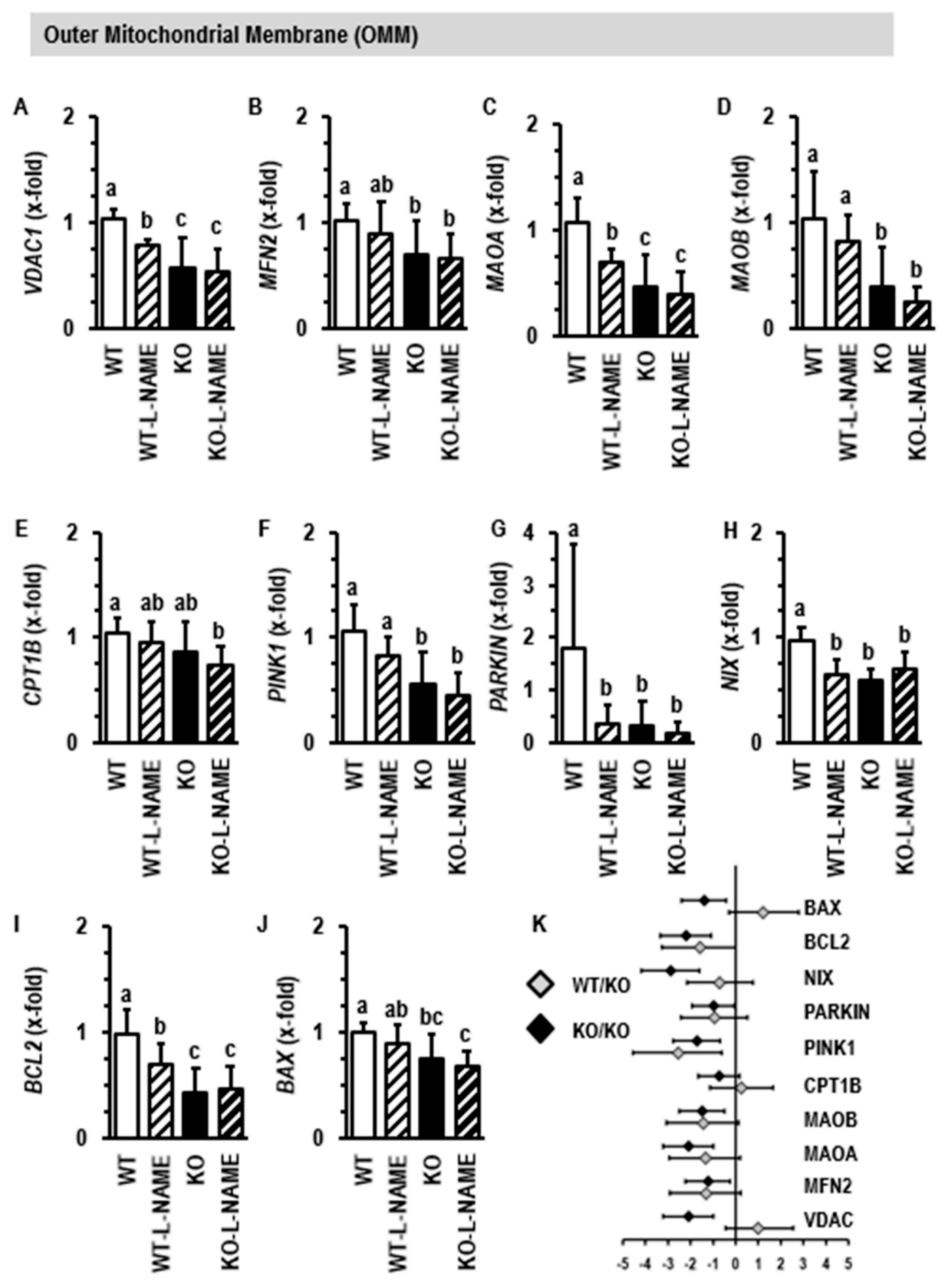
Effect of L-NAME and UCP2 depletion on the expression of genes coding for proteins of the outer mitochondrial membrane (**A**–**J**). Data are means ± S.D. from n = 10 rats per group. (**K**) Gene dose effect on the regulation of genes by depletion of UCP2. Data are effect sizes with confidence intervals. Small different letters indicate differences between other groups with *p* < 0.05 (one-way ANOVA with Student–Newman–Keuls post hoc analysis).

UCP2 depletion also leads to the down-regulation of genes coding for proteins located in the outer mitochondrial membrane. These include *VDAC1*, coding for the protein voltage-dependent anion channel 1 (Figure 9A); *MFN2*, coding for the protein mitofusin 2 (Figure 9B); and *MAOA* and *MAOB*, coding for monoamine oxidase 1 and 2 (Figure 9C,D); but not *CPT1B*, coding for the protein carnitine palmitoyltransferase 1B (Figure 9E). Genes coding for proteins involved in mitochondrial quality control such as *PINK1* coding for *PTEN*-induced kinase 1 (Figure 9F), *PARKIN* coding for the ubiquitin E 3 ligase (Figure 9G), *NIX* coding for the protein NIX (Figure 9H), *BCL2* coding for the protein B-cell lymphoma 2 (Figure 9I), and *BAX* coding for the Bcl2-associated X protein (Figure 9J) were more strongly affected in UCP^−/−^ than in UCP^+/−^ (Figure 9K) and were also affected by L-NAME. Two-way ANOVA identified *NIX* and *BCL2* as genes that are affected by the interaction between UCP2 deletion and hypertension (Supplementary Table S2).

### 3.6. Effect of UCP2 Depletion on Protein Expression

**Figure 10:**
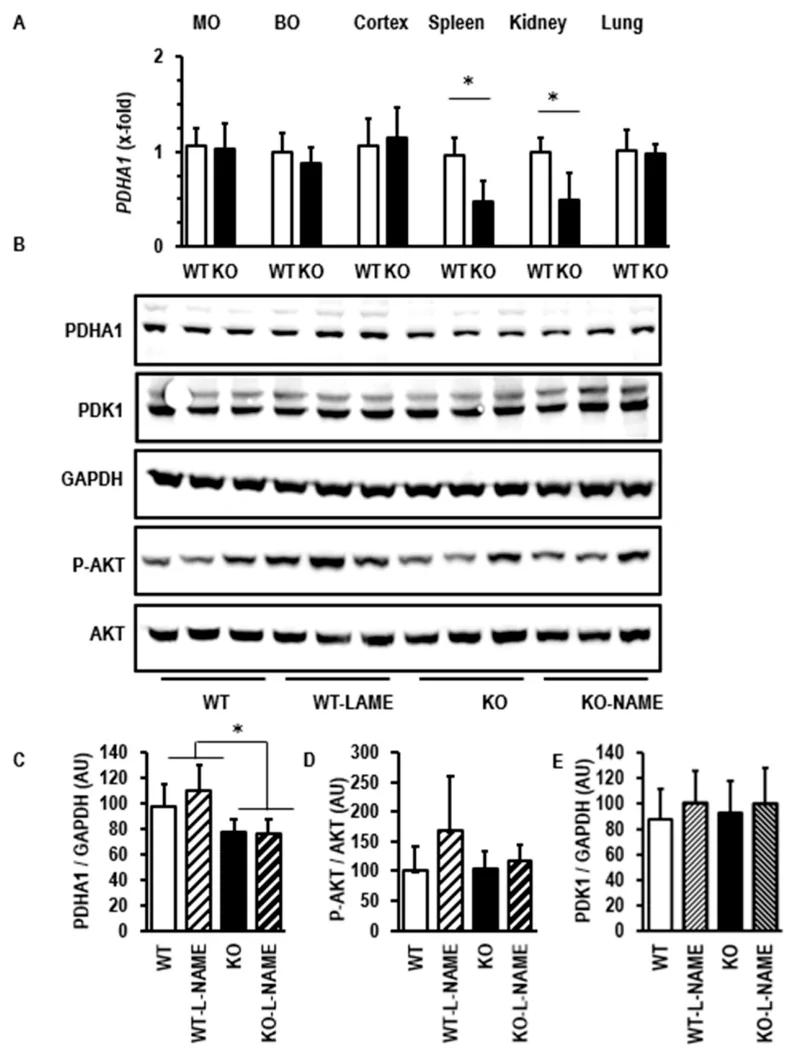
Effect of L-NAME and UCP2 depletion on the expression of pyruvate dehydrogenase E1 subunit alpha (PDHA1). (**A**) Gene expression in various tissues (MO = medulla oblongata; BO = bulbus olfactorius; Cortex = neurocortex). Data are means ± S.D. from n = 8 rats. *, *p* < 0.05 (*t*-Test; n = 8). (**B**) Western Blots showing the protein expression of PDHA1, glycerin aldehyde phosphate dehydrogenase (GAPDH), AKT (Proteinkinase B), and its phosphorylated form (P-AKT). (**C**–**E**) Quantification of the Western Blots shown in B. Data are means ± S.D.; *, *p* < 0.05 (One-Way ANOVA and Student-Newman-Keuls posthoc analysis).

The data suggest that depletion of UCP2 in the spleen is associated with down-regulation of *PDHA1*, coding for a key enzyme in oxidative glucose metabolism. This effect is rather tissue-specific, as it was found also in the kidney but not in the brain or lung (Figure 10A). We next validated that down-regulation of *PDHA1* is accompanied by a corresponding down-regulation of the protein as well. The Western Blot shown in Figure 10B and its quantification shown in Figure 10C indeed confirm that in the absence of UCP2, L-NAME reduced the expression of the protein. The AKT pathway is mainly involved in cell survival. We tested the hypothesis that the depletion of UCP2 in the spleen leading to larger spleen sizes will be supported by the activation of the AKT pathway. As the activation of the AKT pathway requires the phosphorylation of AKT, we analyzed the phosphorylation of AKT (Figure 10B,D). However, we did not see UCP2-dependent activation of the AKT pathway. In contrast to the reduced expression of *PDHA1* in UCP2^−/−^ rats, the inhibitor of the pyruvate dehydrogenase *PDK1* was down-regulated on the level of mRNA but not on the level of protein. Reduced expression of *PDHA1* and normal expression of its inhibitor inhibits oxidative glucose consumption even better. Two-way ANOVA identified PDHA1 as a genotype-dependent regulated gene (Supplementary Table S2).

### 3.7. Effect of UCP2 Depletion on Inflammatory Markers

**Figure 11:**
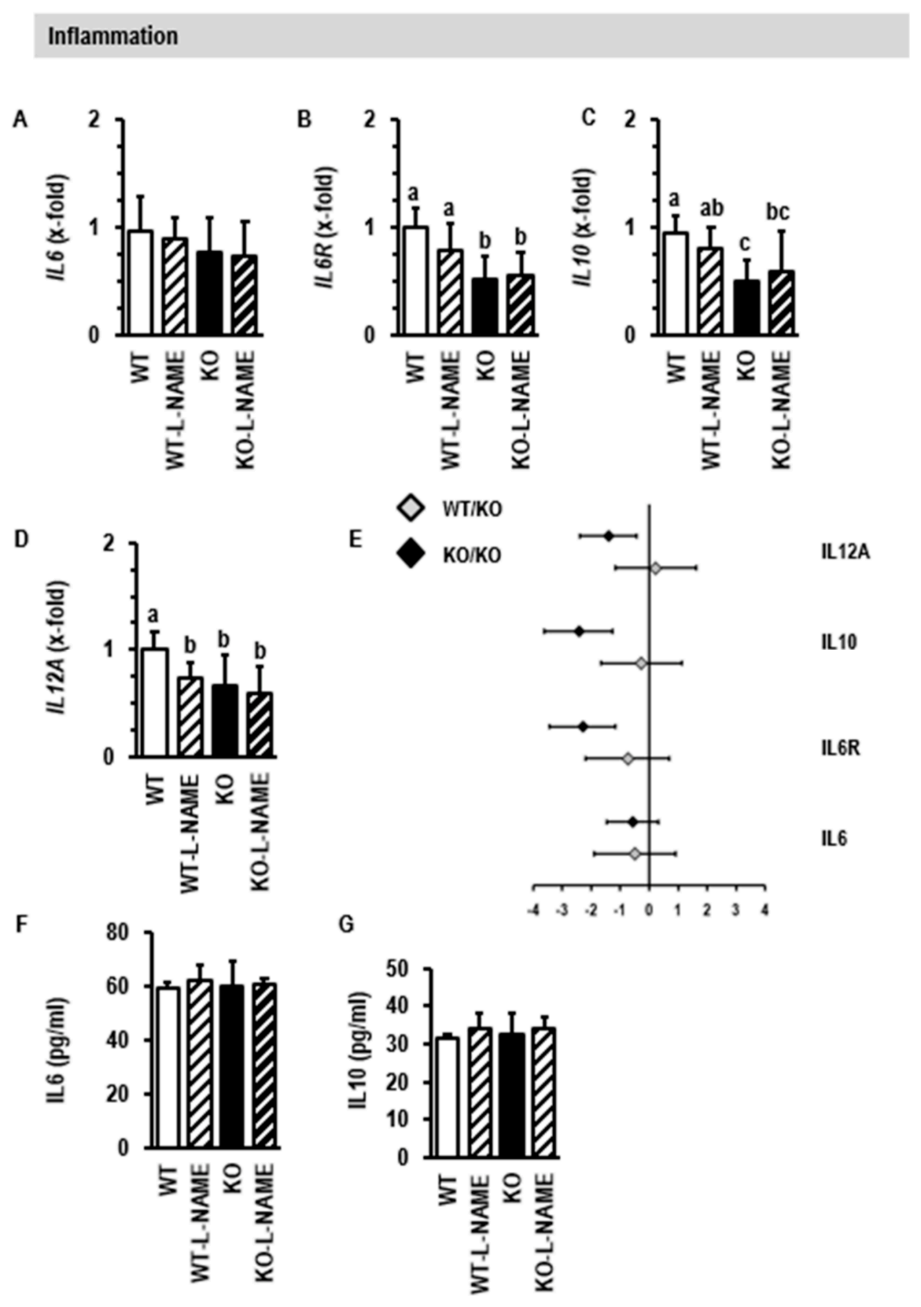
Effect of L-NAME and UCP2 depletion on inflammation in the spleen. (**A**–**D**): Gene expression given as means ± S.D. of n = 8 rats. Small different letters indicate differences between other groups with *p* < 0.05 (one-way ANOVA with Student–Newman–Keuls post hoc analysis. (**E**) Gene dose effect on the regulation of genes by depletion of UCP2. Data are effect sizes with confidence intervals. (**F**,**G**) Plasma concentration of IL6 and IL10 given as means ± S.D. (*p* > 0.05; One-way ANOVA and Student–Newman–Keuls post hoc analysis).

As the spleen acts as a hub connecting the immune system to cardiovascular disease, we finally investigated whether the aforementioned changes in splenic size and expression under depletion of UCP2 and induction of hypertension affect the expression and concentration of inflammatory cytokines. On the gene level, we found no alteration in the splenic expression of *IL6* but a down-regulation of the *IL6R* and of *IL10* (Figure 11A–C). *IL12A*, coding for a cytokine required for natural killer (NK) cell activation in the spleen, was also down-regulated (Figure 11D). The observed alterations showed a stronger effect in UCP^−/−^ than in UCP2^+/−^ rats (Figure 11E). However, plasma concentrations of either IL6 or IL10 were not affected by the different expressions of IL10 in the spleen (Figure 11F,G). Two-way ANOVA identified *IL6R*, *IL10*, and *IL12A* as genotype-dependent regulated genes (Supplementary Table S2).

## 4. Discussion

UCP2 is a mitochondrial protein located at the inner mitochondrial membrane. The precise function of this protein is not yet clear and may differ in various tissues. One possibility is that UCP2 affects mitochondrial-dependent generation of ROS. Another option is that UCP2 fine-tunes the use of different fuels in cells. As UCP2 shows strong expression in the spleen, genetic depletion of UCP2 in rats should affect spleen functions more strongly than functions of other organs with lower expressions of UCP2. Here we show that genetic depletion of UCP2 in rats goes along with an enlarged spleen (splenomegaly). The data suggest a hyperactivity of the organ, which normally means that too many red blood cells and thrombocytes are stored in the spleen and degraded. Our study is the first study showing that UCP2 depletion affects spleen sizes. The spleen is a hub connecting the nervous and immune systems to cardiovascular disease. Interestingly, we found that depletion of UCP2 in rat spleens went along with down-regulation of *NOS3*. Moreover, our data on L-NAME-treated rats show that inhibition of nitric oxide production is sufficient to mimic various aspects of UCP2 depletion in spleen function. One possible explanation for this finding is that UCP2^−/−^ is associated with down-regulation of *NOS3*. In line with this assumption, no additive effects were seen by L-NAME treatment or UCP2 depletion. A link between reduced UCP2 expression and down-regulation of eNOS might be the increase in oxidative stress in the spleen of KO rats. It is well-documented that oxidative stress reduces the expression of eNOS [18].

As this is the first study investigating the spleen of UCP2-depleted rats, it was important to start with a couple of control experiments. First, we confirmed in wild-type littermates of our UCP2^−/−^ colony that the spleen shows the strongest expression of *UCP2* in comparison with other tissues. A high splenic expression of UCP2 has been described before [2,3,4,5,6]. Next, we also confirmed that the splenic expression of *UCP3* is much lower than that of *UCP2*. We then showed that genetic depletion of UCP2 does not lead to a compensatory up-regulation of *UCP3*. This suggests different functions for the two proteins in splenocytes. Finally, we confirmed that depletion of *UCP2* nullified UCP2 protein expression. These control experiments showed that UCP2^−/−^ rats are a suitable model to investigate the role of UCP2 in the rat spleen.

In line with these findings that the spleen has the strongest expression of UCP2 in the body, genetic depletion of UCP2 had a strong and even macroscopically visible effect on the spleen size. This was quantified by weighing of the organ and normalization of the spleen weight to either body weight or tibia lengths. Splenomegaly was also found in a model of chronic colitis that could be affected by exercise [19]. In patients with pulmonary hypertension and right-ventricular hypertrophy, splenomegaly is a common finding [15]. It must be kept in mind that L-NAME treatment of rats can induce right-heart failure [14,16]. This suggests that the overlap between L-NAME and UCP2-deficiency seen here is part of whole-body adaptation to right-heart failure. Furthermore, splenic eNOS signaling is impaired in cirrhotic rats, and rats with portal-hypertension also have enlarged spleen sizes [20,21]. Our findings in the L-NAME rat model and clinical findings suggest that NO represses splenic growth.

In contrast, a recent study on UCP3-deficient mice indicated reduced spleen size [22]. This discrepancy again indicates different physiological functions for UCP2 and UCP3 in the spleen.

Based on sequence homology to UCP1, it is often suggested that UCP2 plays a similar role as UCP1 and acts mainly as an uncoupling protein. However, acting as an uncoupling protein should result in less oxygen consumption in UCP-depleted mitochondria. This was, however, not the case. Moreover, depletion of UCP2 did not modify mitochondrial-derived ROS formation. Collectively, these data do not support the hypothesis that UCP2 affects the electron transport chain, electron transport velocity, or the proton gradient. Therefore, uncoupling seems not to be the main function of UCP2 in splenic mitochondria. Such findings have been described before in lung and spleen tissues [4]. In summary, an important role for UCP2 in the spleen is evident, but its precise function has yet to be identified and seems to be independent of uncoupling function.

We further analyzed the activation of the AKT pathway in these rats. Activation of the AKT pathway in the spleen by administering HgCl_2_ led to smaller spleens and autophagic death triggered by mitochondria [23]. In contrast, induction of immunotoxicity in mouse spleens reduced AKT activation but induced also autophagy [24]. Based on these contrary findings, it is unclear how AKT activity is involved in spleen function and growth control. We found down-regulation of several genes coding for proteins involved in mitochondria quality control. However, we did not see any activation or inhibition of the AKT pathway in the spleen of UCP2-depleted rats. Therefore, in our model of splenomegaly, AKT activity seems not to be involved.

We screened the expression of forty-six genes that are coding for mitochondrial proteins. A first view showed that nearly 74% of genes under investigation were differentially regulated between KO rats and WT rats. *NOS3* was among the strongest down-regulated genes. Interestingly, this effect was highly specific for the spleen as UCP2 depletion in other organs such as heart, vessels, and kidney did not reduce *NOS3* expression. Moreover, when NOS activity was inhibited by administering L-NAME to the rats, nearly 50% of these genes were similarly regulated in the spleen as by deletion of UCP2 expression alone. This suggests that UCP2 depletion regulates gene expression at least in part via reduced bioavailability of nitric oxide (NO). Our findings that in UCP2-depleted spleens, oxidative stress occurs along with the down-regulation of eNOS, as shown before in other tissues and settings as well, may explain why nearly no additive effects can be seen when UCP2 depletion and L-NAME were combined.

Oxidative metabolism of glucose is regulated on the level of the pyruvate dehydrogenase. Here we found that depletion of UCP2 in the spleen is associated with a strong down-regulation of *PDHA1*, which codes for a protein that is an integrative part of the pyruvate dehydrogenase complex. Down-regulation was confirmed on the level of protein. Consequently, we propose a shift from oxidative glucose consumption, also known as OXPHOS, to aerobic glycolysis. Such a shift is known as the Warburg Effect and allows the usage of pyruvate as a source for amino acid synthesis rather than energy production. This regulation may support splenomegaly found in the KO rats. We analyzed isolated mitochondria from the spleen and found no impairment of oxygen consumption with either malate (complex I)- or succinate (complex II)-driven electron transport. This finding is confirmatory to data reported before [22]. The negligible effect on electron transport chain function in combination with the strong down-regulation of PDHA1 led us to suggest that UCP2 is required for the regulation of fuel consumption in splenic mitochondria such as described before for other tissues [7,8]. In such tissues, UCP2 depletion leads to an improvement of glycolysis and reduction of OXPHOS. This leads to the question of how the brain can scope this problem if UCP2 depletion occurs. However, in several areas of the brain such as the Medulla oblongata, the olfactory bulb, and the cerebrum, as well as in the lung, we did not see a down-regulation of *PDHA1*, whereas in the kidney, *PDHA1* was similarly down-regulated as in the spleen. Obviously, the effect of UCP2 expression on the regulation of glucose metabolism is tissue-dependent. In the spleen, we found a down-regulation of proteins required for transition of glucose-derived pyruvate into the citric acid cycles but lower lactate levels. An improved aerobic glucose metabolism with reduced lactate was found before in heart tissue of UCP2 deficiency as well [7]. Unsaturated fatty acids increase the expression of UCP2 in hepatocytes [25]. We found in this study that the depletion of UCP2 in the spleen down-regulates the expression of *HADHA*, a gene coding for a protein that is part of the trifunctional protein required for fatty acid metabolism. It seems that there is a bidirectional pathway. Increased expression of UCP2 favors fatty acid metabolism, whereas depletion of UCP2 favors glucose metabolism. Nevertheless, direct metabolic flux measurements that directly strengthen this conclusion require future studies.

The relationship between UCP2 expression and oxidative stress is complex. On the level of isolated mitochondria, we show that UCP2 deficiency does not affect oxygen consumption; therefore, it does not affect electron transport velocity, and in line with this, we observed no mitochondria-dependent ROS generation. However, in the whole tissue, we show that UCP2 deficiency goes along with a down-regulation of genes coding for enzymes involved in detoxification of ROS, such as *SOD2*, *CAT*, and *GPX1*. These genes code for proteins that are involved in ROS detoxification. SOD2 is located in mitochondria where it detoxifies superoxide, a by-product of the electron transport chain, by conversion into hydrogen peroxide and oxygen. However, hydrogen peroxide requires further conversion into water and oxygen, a reaction catalyzed by the enzyme catalase. The gene *CAT* codes for catalase. Furthermore, hydrogen peroxide is detoxified by glutathione peroxidase. The gene GPX1 codes for this protein. In summary, the collective down-regulation of these genes will increase the risk of oxidative stress. Collectively, the data suggest that UCP2 deficiency does not directly increase ROS formation under standardized conditions via electron transport chain, but that deficiency of anti-oxidative capacity unbalances the relationship between ROS formation and scavenging, leading to cellular oxidative stress.

Interestingly, the expression of many genes coding for proteins involved in mitochondria quality control was also reduced in UCP2-depleted rats, and some of them such as *BCL2*, *PARKIN*, and *NIX* were also strongly affected by L-NAME. This suggests that nitric oxide is required for mitochondrial quality control in the spleen.

The spleen links the autonomous nervous system with immune responses. Monoamine oxidases (MAO-A and MAO-B) play a major role in this linkage. Reduction of norepinephrine levels in the spleen, as found during ageing, contributes to the decline of NK cell activity and increases the risk of cancer [26]. Monoamine oxidases catabolize catecholamines [27]. Here we describe in the spleen of UCP2-depleted rats a down-regulation of genes encoding for the expression of these two important enzymes. This suggests a compensatory effect counteracting immune senescence.

The spleen is a hub connecting immune systems to cardiovascular disease such as hypertension. The contribution of inflammatory cells to the hypertensive phenotype is well-known. We previously described a role for IL6 in this context [12]. Furthermore, the anti-inflammatory cytokine IL10 was shown to reduce hypertension in rodents [28]. The cytokine IL12 is important for the differentiation of dendritic cells and counteracts the function of IL10 [29]. Here we investigated the expression of *IL6*, *ILRR*, *IL10*, and *IL12A* in the spleen of normotensive UCP2-depleted rats and L-NAME-treated rats with hypertension. Neither *IL6* nor *IL10* expressions were affected by hypertension; however, the mRNA expression of the IL6 receptor was reduced in UCP2-depleted rats as well as the expression of *IL10*. In mice, UCP2 knockout resulted in an increased number of phagocytes in the spleen together with reduced IL10 [30]. In other words, genetic depletion of UCP2 in rats and mice reduced *IL10* expression. In general, correlations between splenic expression of cytokines and plasma concentrations are low, except that of IL10 [31]. We even found no coupling between splenic expression of *IL10* and plasma concentration of IL-10 in UCP2-depleted rats. Furthermore, studies with cholinergic stimulation of the spleen showed anti-inflammatory reactions in normotensive but not hypertensive rats [32,33]. In general, IL10 is considered a cytokine that transmits splenic adaptation to stress to other organs such as the heart and kidney, but the lack of reduced plasma IL10 does not support such a mechanism in UCP2-depleted rats [34]. L-NAME and UCP2 depletion also showed a reduced expression of *IL12A*. The function of this cytokine is not well-understood. Nevertheless, based on this finding, a modification of immune response in hypertension cannot be excluded, and again UCP2-dependent processes seem to be part of this. In any case, future studies must focus on the characterization of splenic cell composition, but this was out of scope of this study. In more detail, it must be clarified whether the reduced expression of IL10, an anti-inflammatory cytokine, in spleens from UCP2-deleted cells indicates a local effect or must be seen in the light of inter-organ communication.

## 5. Conclusions

This is the first description of the effect of *UCP2* depletion on the rat spleen. The data first show a macroscopically obvious phenotype. The fact that the spleen is primarily affected, but no other organs, might be explained by the fact that the spleen displays the highest expression of *UCP2*. Secondly, the down-regulation of *PDHA1* suggests a Warburg-like phenotype that favors cell growth. Third, we recognized that the down-regulation of *NOS3* in UCP2-depleted spleens and pharmacological inhibition of NOS induced a similar phenotype than in UCP2-depleted rats. Furthermore, the lack of additive effects of L-NAME administration and *UCP2* depletion supports the view that the UCP2-dependent regulation of *NOS3* triggers some of the observed findings. Finally, the different expression of *IL12A* in the spleen at least suggests a role for UCP2/NOS in the coupling between the immune system and hypertension. Therefore, the newly generated UCP2-depleted rat seems to be a suitable model to improve our understanding how the spleen contributes to cardiovascular diseases.

## Data Availability

The original contributions presented in this study are included in the article/Supplementary Material. Further inquiries can be directed to the corresponding author.

## Supplementary Materials

The following supporting information can be downloaded at: https://www.mdpi.com/article/10.3390/cimb48080775/s1.

## Abbreviations

The following abbreviations are used in this manuscript:

L-NAME	L-N^G^-Nitro arginine methyl ester
NOS	Nitric oxide synthase
ROS	Reactive oxygen species
UCP	Uncoupling Protein
