# Supplementary material for: Uncoupling Protein 2 and Nitric Oxide Deficiency Favor Glycolytic Pathways and Organ Growth in the Rat Spleen
Source: Curr Issues Mol Biol. 2026 Jul 30;48(8):775. doi: 10.3390/cimb48080775 (PMC13510864; doi:10.3390/cimb48080775)
Supplement: Supplementary file 1 [file cimb-48-00775-s001.zip › Supplement Materials.pdf]

Supplement Table S1: List of primers used for PCR

| Gene name | forward | reverse | NCBI Ref. Seq. |
|---|---|---|---|
| ARG1 | GGAAGCATCTCTGGCCACGCC | CACCGGTTGCCCGTGCAGAT | NC_086019.1 |
| ARG2 | TGAGGAGCAGCGTCTCCCGT | GCTTCTCGGATGGCGGCTGG | NC_086024.1 |
| B2M | GCCGTCGTGCTTGCCATTC | CTGAGGTGGGTGGAACTGAGA C | NM_017087 |
| BAX | ACTAAAGTGCCCGAGCTGATC | CACTGTCTGCCATGTGGGG | NM_017059 |
| BCL2 | ATCTTCTCCTTCCAGCCTGA | TCAGTCATCCACAGAGCGAT | NM_016993 |
| BNIP3L | CTCACTTAGTCGAGCCGCCG | GCCATTGCTGCTGTTCATGG | NM_080888.2 |
| CAT | TTTTCACCGACGAGATGGCA | CTGACTCTCCAGCGACTGTG | NM_012520 |
| CD36 | GGCTCATTACTGGAGCCGTT | TACACAGTGGTGCCCGTTTT | NM_031561.2 |
| COL1A1 | GCGAACAAGGTGACAGAG | CCAGGAGAACCAGCAGAG | NM_053304 |
| COL3A1 | TGGAGTCGGAGGAATG | GCCAGATGGACCAATAG | NM_032085.1 |
| CPT1B | GACCCAAAGCAGTACCCCAA | GCAGGGCGTTCGTTTCTGA | NM_013200.2 |
| DNM1 | TGGAAAGAGCTCAGTGCTGG | TCAACTCCATTTTCTTCTCCTGT | NM_053655.3 |
| FABP3 | TGGAAGCTAGTGGACAGCAAG | GATTGTGGTCGGCTTGGTCA | NM_024162.2 |
| FABP5 | GGCAGGAAAACTGAGACGGT | TGGCATTGTTCATGACGCAC | NM_145878.2 |
| FN1 | TGGAGCAAGAAGGACAAC | CGGACATCTGTGAAGGAG | NM_019143 |
| GPX1 | AGTGCGAGGTGAATGGTGAG | GGAACACCGTCTGGACCTAC | NM_030826 |
| HADHA | ACATCGGAGCAGTCTTTGGG | AGCTGACACGGGGTAAACTG | NM_130826.2 |
| HIF1A | ATCACTGTCACTGCCACCG | GCTGTCCGACTGTGAGTACC | NM_024359.1 |
| HIF2A | GGGTACGTGAGGCATGTTGA | CCGTCGGTCAGACCAGAAAA | NM_001004083.1 |
| HPRT | CCAGCGTCGTGATTAGTGAT | CAA GTC TTT CAG TCC TGT CC | NM_012583 |
| IL6 | CACTTCACAAGTCGGAGGCT | TCTGACAGTGCATCATCGCT | NM_012589.2 |
| IL6R | TGGTCAAAGACGTTCACGGT | CTCTGCTAGCCAAGGAGTGC | NM_017020.3 |
| IL10 | CCTCTGGATACAGCTGCGAC | TGAGTGTCACGTAGGCTTCT | NM_012854.2 |
| IL12A | TGTGTCAATCACGCTACCTCC | AAGACACTTGGCAGGTCCAG | NM_053390.1 |
| LAMA1 | CGAGGATGTCAGCGTTGTC | TCACAGCCGTCTCCAGTC | NM_012974 |
| LAMP2 | AAAGAGCAGGTGGTTTCCGT | TAAGTACTCCTCCCAGGGCT | NM_017068.3 |
| LDHC | AGCCTTTTCCTCAGCACTCC | TGTTTTGGATTACGCCGGGA | NM_017266.2 |
| MAOA | GTTGGACAAAAATGCTCGGG | GGAACCCACAGGGCAGATACC | NM_033653.1 |
| MAOB | GGCTGTGCTGCTATAATGFFA | ATAATGGACTGGCTGCAGAGC | NM_013198.1 |
| MAP1LC3A | TTGGTCAAGATCATCCGGCG | AGCCGAAGGTTTCTTGGGAG | NM_199500.2 |
| MFN2 | CTCTGTGCTGGTTGACGAGT | AGCGGTCAGACATGTTTCGT | NM_130894.4 |
| MPC1 | ATGTCCGGAGCAAGGACTTC | GAAAGTCATCCGCCCACTGA | NM_133561.2 |
| MPC2 | GTGCTGATGGCTACAGGGTT | ACTGGATTCCTTTAGATTTGAGTTC | NM_001077643.2 |
| NOS3 | AGCCCGGGACTTCATCAATCAG | GCCCCAAACACCAGCTCACTCTC | NM_021838 |
| OMA1 | TAGCGTGACCGATATGCACC | CTGCCAAGCTGTCTCTTGGA | NM_012615 |
| PARK2 | GGCCTCCAAGGAAACCATCA | TCCACTCACAGCCACAGTTC | NM_001004072.2 |
| PDHA1 | CAGAAGCCGGCAAGCAG | ATACAGCTGATCCGCCTTGAG | NM_001004072.2 |
| PDK1 | GCCAGGTGGACTTCTATGCG | TGTACGGATGGGGTCCTGAG | NM_053826.2 |
| PINK1 | TGTATGAAGCCACCATGCCC | TCTGCTCCCTTTGAGACGAC | NM_001106694.1 |
| PPARA | TGCGACATCATGGAACCCAA | CACAATCCCCTCCTGCAACT | NM_013196.2 |
| SLC2A1 | GCTGTGGCTGGCTTCTCTAA | CCGGAAGCGATCTCATCGAA | NM_138827.1 |
| SLC2A4 | ACCGTCTTCACGTTGGTCTC | ATCAAGATGGCACAGCCACA | NM_012751.1 |
| SOD1 | AAGGCCGTGTGCGTGCTGAA | ACATGGAACCCATGCTCGCCT | NM_017050 |
| SOD2 | ATGTTGTGTCGGGCGGCGTG | TCGCGTGGTGCTTGCTGTGG | NM_017051 |
| SOD3 | TGCTGCCTCCCGATCAGCCA | CCCTGGCTCAGGTCCCCGAA | NM_012880 |
| UCP2 | CACCGTCATTGCCTCCCCCG | CGGAGCATGGTCAGGGCACA | NM_019354.2 |
| UCP3 | GATCTCCTCACCTTCCCCCT | AGGCAAAACTCATCTGGCGA | NM_013167.2 |
| VDAC | CTACGGGCTCATCTTCACCC | TGTCAACGTTACTGCCCACA | XM_006253358.1 |

Supplement Table S2: Two-Way ANOVA for gene expression in the spleen

| Gene | Genotype | Treatment | Interaction |
|---|---|---|---|
| Mitochondrial Matrix (Fig. 6) | | | |
| *PDHA1* | p<0.001 | p=0.149 | p=0.024 |
| *PDK1* | p<0.001 | p=0.313 | p=0.381 |
| *HADHA* | p<0.001 | p=0.023 | p=0.673 |
| *SOD2* | p<0.001 | p=0.263 | p=0.319 |
| *GPX1* | p<0.001 | p=0.019 | p=0.105 |
| *CAT* | p<0.001 | p=0.001 | p=0.001 |
| *ARG2* | p=0.421 | p=0.716 | p=0.083 |
| Inner Mitochondrial Membrane (Fig. 8) | | | |
| *UCP2* | p<0.001 | p=0.161 | p=0.195 |
| *UCP3* | p=0,136 | p=0.998 | p=0.954 |
| *OMA1* | p<0.001 | p=0.201 | p=0.723 |
| *MPC1* | p<0.001 | p=0.040 | p=0.100 |
| *MPC2* | p<0.001 | p=0.183 | p=0.972 |
| Outer Mitochondrial Membrane (Fig. 9) | | | |
| VDAC1 | p<0.001 | p=0.029 | p=0.082 |
| MFN2 | p=0.004 | p=0.415 | p=0.654 |
| MAOA | p<0.001 | p=0.006 | p=0.060 |
| MAOB | p<0.001 | p=0.117 | p=0.713 |
| CPT1B | p=0.009 | p=0.153 | p=0.827 |
| PINK1 | p<0.001 | p=0.054 | p=0.429 |
| PARKIN | p=0.025 | p=0.030 | p=0.060 |
| NIX | p=0.001 | p=0.033 | p<0.001 |
| BCL2 | p<0.001 | p=0.099 | p=0.039 |
| BAX | p<0.001 | p=0.100 | p=0.711 |
| Superoxide formation (Fig. 7) | | | |
| DHE Staining | p<0.001 | p<0.001 | p<0.001 |
| Protein Expression (Fig. 10) | | | |
| PDHA1/GAPDH | p<0.001 | p=0.294 | p=0.365 |
| PAKT/AKT | p=0.364 | p=0.116 | p=0.277 |
| PDK/GAPDH | p=0.841 | p=0.386 | p=0.874 |
| Inflammation (Fig. 11) | | | |
| IL6 | p=0.074 | p=0.589 | p=0.898 |
| IL6R | p<0.001 | p=0.239 | p=0.112 |
| IL10 | p<0.001 | p=0.753 | p=0.158 |
| IL12A | p<0.002 | p=0.024 | p=0.153 |
| ELISA (Fig. 11) | | | |
| IL6 | p=0.962 | p=0.081 | p=0.114 |
| IL10 | p=0.738 | p=0.329 | p=0.853 |

p values are raw data not corrected for multiple corrections

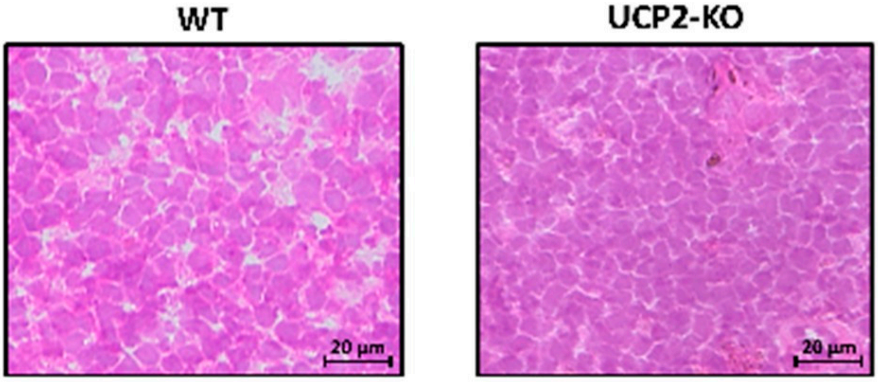


**Supplementary Figure S1.** A larger magnification of Figure 2G.
